# Relationship between Plasma and Intracellular Concentrations of Bedaquiline and Its M2 Metabolite in South African Patients with Rifampin-Resistant Tuberculosis

**DOI:** 10.1128/AAC.02399-20

**Published:** 2021-10-18

**Authors:** Precious Ngwalero, James C. M. Brust, Stijn W. van Beek, Sean Wasserman, Gary Maartens, Graeme Meintjes, Anton Joubert, Jennifer Norman, Sandra Castel, Neel R. Gandhi, Paolo Denti, Helen McIlleron, Elin M. Svensson, Lubbe Wiesner

**Affiliations:** a Division of Clinical Pharmacology, Department of Medicine, University of Cape Town, Cape Town, South Africa; b Albert Einstein College of Medicine & Montefiore Medical Center, Bronx, New York, USA; c Department of Pharmacy, Radboud Institute for Health Sciences, Radboud University Medical Center, Nijmegen, The Netherlands; d Wellcome Centre for Infectious Diseases Research in Africa, Institute of Infectious Disease and Molecular Medicine, University of Cape Town, Cape Town, South Africa; e Department of Medicine, University of Cape Town, Cape Town, South Africa; f Rollins School of Public Health and Emory School of Medicine, Emory University, Atlanta, Georgia, USA; g Department of Pharmacy, Uppsala University, Uppsala, Sweden

**Keywords:** drug-resistant tuberculosis, bedaquiline, metabolite, intracellular, pharmacokinetics

## Abstract

Bedaquiline is recommended for the treatment of all patients with rifampin-resistant tuberculosis (RR-TB). Bedaquiline accumulates within cells, but its intracellular pharmacokinetics have not been characterized, which may have implications for dose optimization. We developed a novel assay using high-performance liquid chromatography-tandem mass spectrometry (LC-MS/MS) to measure the intracellular concentrations of bedaquiline and its primary metabolite M2 in patients with RR-TB in South Africa. Twenty-one participants were enrolled and underwent sparse sampling of plasma and peripheral blood mononuclear cells (PBMCs) at months 1, 2, and 6 of treatment and at 3 and 6 months after bedaquiline treatment completion. Intensive sampling was performed at month 2. We used noncompartmental analysis to describe plasma and intracellular exposures and a population pharmacokinetic model to explore the relationship between plasma and intracellular pharmacokinetics and the effects of key covariates. Bedaquiline concentrations from month 1 to month 6 of treatment ranged from 94.7 to 2,540 ng/ml in plasma and 16.2 to 5,478 ng/ml in PBMCs, and concentrations of M2 over the 6-month treatment period ranged from 34.3 to 496 ng/ml in plasma and 109.2 to 16,764 ng/ml in PBMCs. Plasma concentrations of bedaquiline were higher than those of M2, but intracellular concentrations of M2 were considerably higher than those of bedaquiline. In the pharmacokinetic modeling, we estimated a linear increase in the intracellular-plasma accumulation ratio for bedaquiline and M2, reaching maximum effect after 2 months of treatment. The typical intracellular-plasma ratios 1 and 2 months after start of treatment were 0.61 (95% confidence interval [CI]: 0.42 to 0.92) and 1.10 (95% CI: 0.74 to 1.63) for bedaquiline and 12.4 (95% CI: 8.8 to 17.8) and 22.2 (95% CI: 15.6 to 32.3) for M2. The intracellular-plasma ratios for both bedaquiline and M2 were decreased by 54% (95% CI: 24 to 72%) in HIV-positive patients compared to HIV-negative patients. Bedaquiline and M2 were detectable in PBMCs 6 months after treatment discontinuation. M2 accumulated at higher concentrations intracellularly than bedaquiline, supporting *in vitro* evidence that M2 is the main inducer of phospholipidosis.

## TEXT

With more than 500,000 new cases each year, multidrug-resistant tuberculosis (MDR-TB)—defined as resistance to at least isoniazid and rifampin—continues to undermine global TB control (1). MDR-TB is more difficult to treat than drug-susceptible TB and is associated with substantially worse outcomes. The anti-TB drug bedaquiline, approved in 2012 by the Food and Drug Administration (FDA), significantly improves MDR-TB treatment outcomes (2). The World Health Organization (WHO) now categorizes bedaquiline as a group A medicine and recommends that it be included in the regimen for all patients with rifampin-resistant TB (RR-TB) and MDR-TB (3).

Mycobacterium tuberculosis is an intracellular pathogen. The efficacy of antituberculosis agents and the propensity for selection of resistance to them are related to their intracellular concentrations (4, 5). Because intracellular drug concentrations are difficult to measure, plasma concentrations are typically used as a surrogate, but plasma concentrations of many drugs may not mirror those intracellularly (6). Bedaquiline distributes extensively into the tissues with an estimated volume of distribution of >10,000 liters at steady state (7). Both bedaquiline and its primary metabolite, M2, are known to have intracellular antimycobacterial activity (8–11), but the intracellular concentrations of both are unknown. Understanding intracellular pharmacokinetics (PK) of bedaquiline and M2 could help define exposure-efficacy relationships, ultimately informing dose optimization for this essential drug.

Like other cationic amphiphilic drugs, bedaquiline induces phospholipidosis in the cells it penetrates (12, 13). Although the clinical significance of phospholipidosis remains controversial, drugs inducing phospholipidosis have been associated with QT prolongation. *In vitro* studies suggest that M2 is a stronger inducer of phospholipidosis than the parent drug (14, 15). Although M2 circulates at a 10-fold-lower concentration in plasma than the parent drug (14, 16), it is thought to drive QT prolongation associated with bedaquiline use (17). Understanding how M2 concentrates in cells may help elucidate its contribution to toxicity (14, 16).

In this study, we measured bedaquiline and M2 concentrations in plasma and peripheral blood mononuclear cells (PBMCs) using high-performance liquid chromatography-tandem mass spectrometry (LC-MS/MS) at intervals during and after treatment for RR-TB. Our objective was to describe the intracellular pharmacokinetics of bedaquiline and M2 in patients with drug-resistant TB and the relationship between plasma and intracellular concentrations of both.

## RESULTS

### Study population and sparse pharmacokinetic sampling.

The baseline characteristics of the 21 enrolled participants are summarized in Table 1. Pharmacokinetic data were available for plasma bedaquiline and its M2 metabolite at month 1 (*n* = 18), month 2 (*n* = 20), and month 6 (*n* = 11) of treatment and at month 3 (*n* = 2) and month 6 (*n* = 1) after stopping bedaquiline. For intracellular bedaquiline and M2, pharmacokinetic data were available for month 1 (*n* = 21), month 2 (*n* = 18), and month 6 (*n* = 9) of treatment and at 3 months (*n* = 3) and 6 months (*n* = 2) after bedaquiline treatment completion.

**TABLE 1:** Baseline characteristics of the participants

Characteristic^*a*^	Value for participants (*n* = 21)
Age (yrs), median (IQR)	29 (27–46)
Female sex, no. (%)	12 (57)
Race Mixed race, no. (%) Black, no. (%)	15 (71) 6 (29)
BMI (kg/m^2^), median (IQR)	19.8 (17.9–26.7)
Body wt (kg), median (IQR)	54 (44.3–63.4)
Serum creatinine (μmol/liter), median (IQR)	52.5 (48.3–57.0)
eGFR (ml/min), median (IQR)	116.6 (108.7–127.9)
HIV positive, no. (%)	10 (48)
Receiving lopinavir-ritonavir-based ART, no. (% HIV positive)	5 (50)
Receiving nevirapine-based ART, no. (% HIV positive)	5 (50)

a Abbreviations: IQR, interquartile range; BMI, body mass index; eGFR, estimated glomerular filtration rate, derived from Cockcroft-Gault formula; ART, antiretroviral therapy.

**FIG 1:**
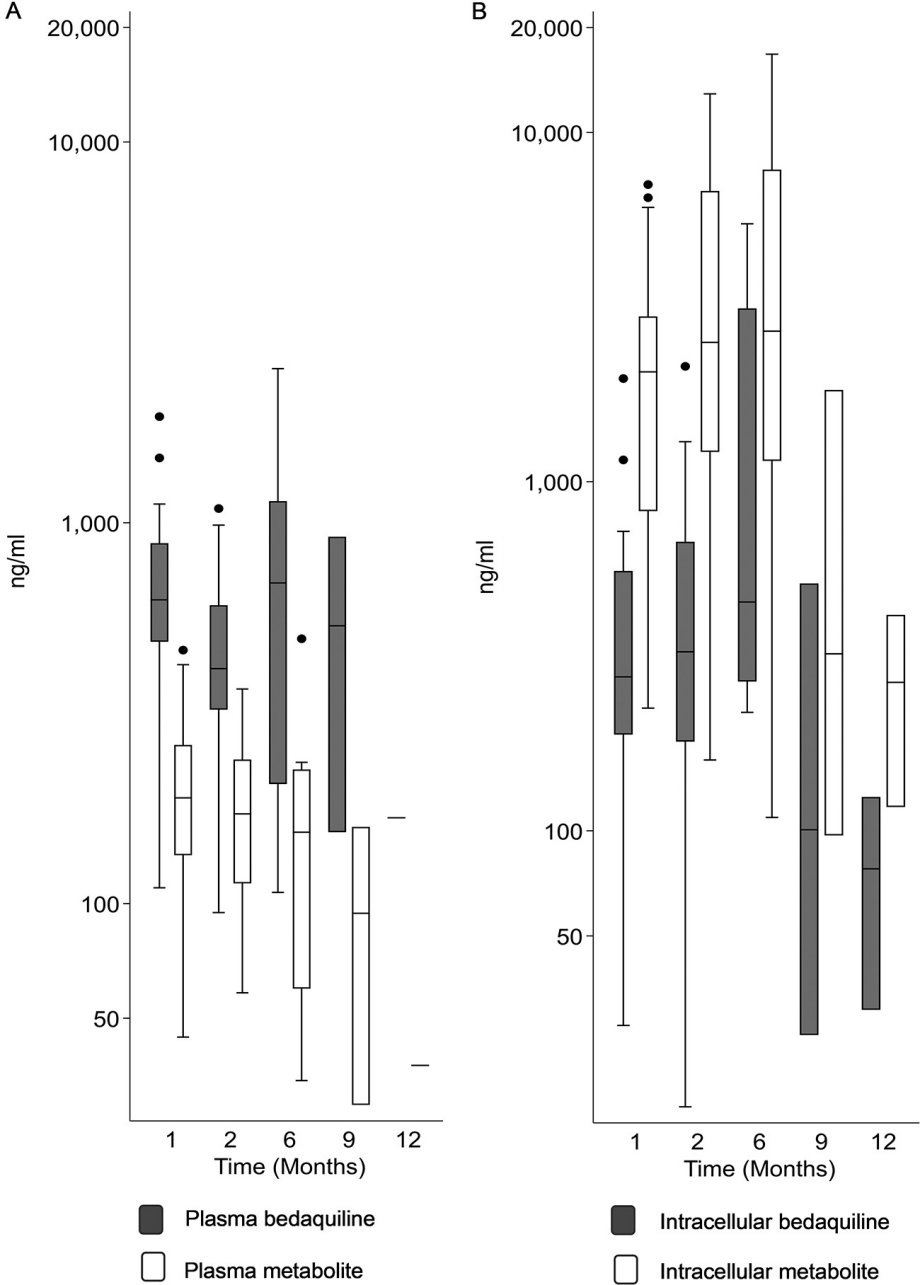
Observed plasma (A) and intracellular (B) bedaquiline and M2 concentrations from sparse sampling during and after (month 9 and month 12) bedaquiline treatment.

Plasma and intracellular bedaquiline and M2 concentrations at the different time points are shown in Fig. 1. Among participants who had paired plasma and intracellular measurement data from at least one visit, median intracellular concentrations of bedaquiline were significantly lower than the plasma concentrations after 1 month of bedaquiline treatment (277 ng/ml versus 628 ng/ml; *P* = 0.01; *n* = 18). However, no significant difference was observed between intracellular and plasma bedaquiline concentrations at month 2 (326 ng/ml versus 450 ng/ml; *P* = 0.37; *n* = 18) or at month 6 (912 ng/ml versus 719 ng/ml; *P* = 0.21; *n* = 8). The median intracellular concentrations of M2 were significantly higher than the plasma concentrations at month 1 (2,252 ng/ml versus 190 ng/ml; *P* < 0.01; *n* = 18), month 2 (2,506 ng/ml versus 185 ng/ml; *P* < 0.01; *n* = 18), and month 6 (4,346 ng/ml versus 147 ng/ml; *P* = 0.01; *n* = 8).

### Intensive pharmacokinetic sampling.

**FIG 2:**
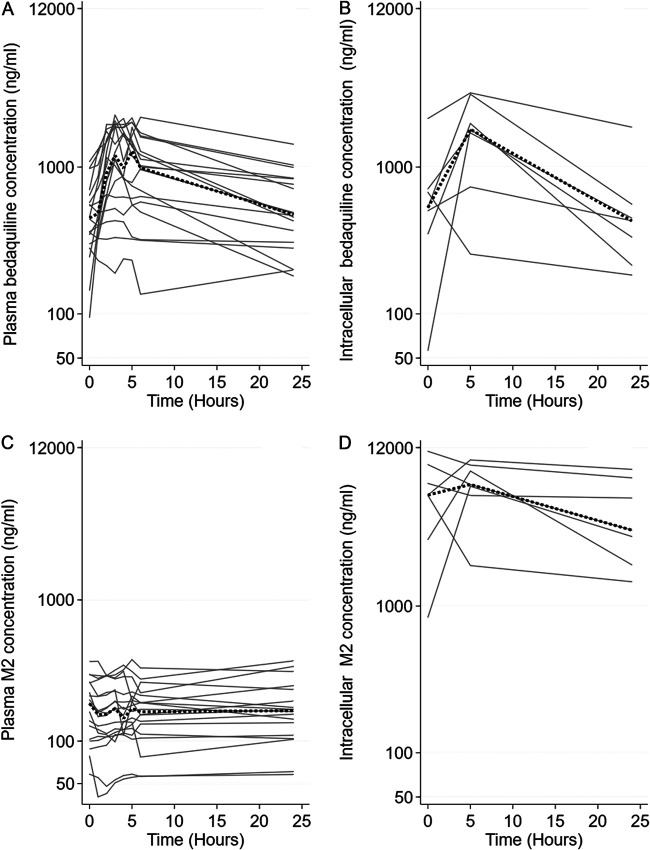
Individual (*n* = 18) plasma concentration-time plots over 24 h at intensive pharmacokinetic sampling, at 2 months of treatment, for plasma bedaquiline (A) and M2 (B) and corresponding concentration-time plots for intracellular bedaquiline (C) and M2 (D) in PBMCs over 24 h (*n* = 7). The dotted line shows the median.

At month 2, 18 participants underwent intensive plasma sampling and those that had complete PK data at all three PBMC sampling time points (*n* = 7) also underwent intensive intracellular sampling to determine bedaquiline and M2 concentrations. We observed high interindividual variability of the pharmacokinetic data (Fig. 2). The coefficients of variation (CV) of the predose trough concentration (*C*_min_) were 50.7% for plasma bedaquiline, 45.2% for plasma metabolite, 94.5% for intracellular bedaquiline, and 82.4% for intracellular metabolite. There was a 2.2-fold increase in bedaquiline plasma concentration from predose to 5 h (*P* < 0.01). Similarly, the intracellular bedaquiline concentrations increased from predose to 5 h (*P* < 0.01) but declined to the predose concentration by 24 h (*P* = 0.78). In contrast, plasma and intracellular M2 concentrations were not found to be significantly different when comparing predose versus 5-h or predose versus 24-h samples (*P* > 0.30).

Among paired samples (*n* = 7), there were no significant differences between plasma and intracellular bedaquiline area under the 24-h concentration-time curve (AUC_0–24_) (*P* = 0.24), the peak concentration (*C*_max_) (*P* = 0.18), or the *C*_min_ (*P* = 0.61). However, intracellular M2 AUC_0–24_, *C*_max_, and *C*_min_ were all significantly higher than in plasma, with *P* values of <0.02 (Table 2).

**TABLE 2:** Pharmacokinetic parameters for plasma and intracellular bedaquiline and M2 during intensive sampling at month 2 of bedaquiline treatment

Pharmacokinetic parameter	Value^*a*^ for:			
	Bedaquiline		M2	
	Plasma	Intracellular	Plasma	Intracellular
AUC_0–24_ (ng/ml·h)	20,115 (11,626–27,398), *n* = 18	26,505 (14,124–44,213), *n* = 7	3,966 (2,664–5,504), *n* = 18	134,937 (108,880–208,621), *n* = 7
*C*_max_ (ng/ml)	1,715 (650–2,060), *n* = 18	1,805 (731–3,132), *n* = 7	192 (135–262), *n* = 18	8,308 (6,544–9,889), *n* = 7
*C*_min_ (ng/ml)	450 (299–642), *n* = 18	529 (350–710), *n* = 7	184 (103–258), *n* = 18	3,287 (2,838–9,191), *n* = 7

a Data presented as medians (IQRs).

### Population pharmacokinetic modeling.

In the population pharmacokinetic analysis, 20 of the 21 participants were included, as 1 patient was missing dosing information. In total, these patients provided 187 plasma pharmacokinetic observations and 67 intracellular pharmacokinetic observations each for bedaquiline and M2. The plasma pharmacokinetic data were fitted well using a previously established model for bedaquiline and M2 (18). Using the individual plasma pharmacokinetic parameters as input, we developed a model for intracellular bedaquiline and M2 pharmacokinetics. The goodness-of-fit plots and code for the intracellular pharmacokinetic model are shown in the supplemental material. The final pharmacokinetic parameters are shown in Table 3. The intracellular-plasma equilibration half-life was fixed at 1 min (i.e., effectively instantaneous distribution), as it could not be estimated reliably and went to infinitely small values. We estimated a linear increase over time on treatment in the intracellular-plasma ratio for bedaquiline and M2, reaching maximum accumulation after about 2 months of treatment (1,500 h). The time to maximum effect was chosen to reflect accumulation during the life span of PBMCs, taking into account the frequencies of different PBMCs and their life spans (19). An exploration of different times to maximum effect showed that this was also a reasonable assumption based on predictive performance. The intracellular-plasma accumulation ratio at month 2 (the maximum of the time effect) was estimated for both bedaquiline and M2, and the ratio at the start of treatment (the intercept of the time effect) was estimated as one fraction of the ratio at month 2 for both bedaquiline and M2. Following this time effect, the typical intracellular-plasma ratios at 1 month after start of treatment were 0.61 (95% confidence interval [CI]: 0.42 to 0.92) for bedaquiline and 12.4 (95% CI: 8.8 to 17.8) for M2. Of the tested covariate effects, HIV coinfection was associated with a statistically significant decrease in intracellular-plasma accumulation ratio. This decreased accumulation ratio was driven by relatively lower intracellular concentrations in HIV-positive patients than in HIV-negative patients. Interindividual variability was shared between the intracellular-plasma accumulation ratios of bedaquiline and M2. The proportional residual errors for the intracellular concentrations of bedaquiline and M2 were estimated to be relatively large.

**TABLE 3:** Final pharmacokinetic parameters for the intracellular model of bedaquiline and M2^*a*^

Parameter	Estimate (SIR 95% CI)
Intracellular-plasma equilibration half-life, min	1, fixed
Maximum intracellular-plasma accumulation ratio bedaquiline (mo 2 and later)	1.10 (0.74–1.63)
Maximum intracellular-plasma accumulation ratio M2 (mo 2 and later)	22.2 (15.6–32.3)
Factor of maximum intracellular-plasma accumulation ratio at start of treatment	0.123 (0.003–0.439)
HIV effect on the intracellular-plasma accumulation ratio	0.46 (0.26–0.76)
Interindividual variability of the intracellular-plasma accumulation ratio, % CV	51.2 (33.1–85.3)
Proportional residual error intracellular bedaquiline, % CV	106 (86.2–139)
Proportional residual error intracellular M2, % CV	80.4 (65.1–103)

a CI, confidence interval; CV, coefficient of variation; SIR, sampling importance resampling.

## DISCUSSION

We determined the concentrations of bedaquiline and its M2 metabolite in plasma and PBMCs, together with the intracellular-to-plasma ratios, over time in HIV-positive and HIV-negative participants with culture-confirmed RR-TB. As expected, plasma concentrations of bedaquiline were higher than those of M2, but intracellular concentrations of M2 were considerably higher than those of bedaquiline. The intracellular-to-plasma concentration ratios of both bedaquiline and M2 increased over time and reached their maximum ratios at month 2 of bedaquiline treatment, indicating intracellular accumulation over time. Both bedaquiline and M2 were detectable in plasma and PBMCs 3 and 6 months following cessation of treatment.

Intracellular drug concentrations play an important role in the efficacy and toxicity of a TB drug (20, 21). Because M. tuberculosis is an intracellular pathogen, our finding that the median intracellular concentration of bedaquiline was above the wild-type MIC (250 ng/ml) (22, 23) is reassuring and is in line with previous studies reporting rapid culture conversion and intracellular bacteriostatic effects at the recommended dose (9, 24). Our observation that bedaquiline and M2 accumulate over time is consistent with binding to intracellular phospholipids (8, 9, 25). It is unknown if this intracellular accumulation results in increased efficacy over treatment time through higher exposure to bedaquiline for intracellular M. tuberculosis. The intracellular accumulation reverses slowly upon treatment discontinuation, resulting in its prolonged terminal elimination half-life (7). We confirmed that both bedaquiline and M2 are detectable in plasma and in PBMCs 3 and 6 months following cessation of treatment, as had been shown in previous studies (9, 18).

Unlike for bedaquiline, intracellular concentrations of M2 were significantly higher in PBMCs than in plasma over the 6-month treatment period. There are several possible explanations for this difference between bedaquiline and M2. In plasma, bedaquiline and M2 are highly protein bound: >99.9% and >99.7%, respectively (11). As the intracellular concentration of a drug or metabolite is determined by its unbound plasma concentration (20), the minimal difference in the protein binding in plasma may contribute to their substantially different intracellular concentrations. Bioavailability is also affected by transport proteins at the cell membrane and intracellular enzymes (26, 27), and higher intracellular concentrations of M2 may be due to differences in substrate specificity for influx and efflux transporters (28–32). Finally, M2 may bind more avidly to intracellular phospholipids, which seems plausible, as *in vitro* studies show that phospholipidosis is driven by M2 (14, 15). It is possible that the higher intracellular concentrations of M2 may explain its greater toxicity, notably QT prolongation (14, 33).

Being HIV positive was associated with a 54% decrease in the intracellular-to-plasma bedaquiline and M2 ratios. The lower relative intracellular concentrations in HIV-positive individuals may be due to HIV infection-related dysfunction of immune cells which affects the transfer of the drug from the plasma into the cells and may also be due to differences in longevity of PBMCs (34, 35). The median albumin concentration during the complete treatment period was 38 g/liter, with similar median concentrations observed for HIV-positive (39 g/liter) and HIV-negative (38 g/liter) individuals. Additionally, lopinavir-ritonavir was the only administered anti-HIV drug known to influence bedaquiline and M2 exposure, but the plasma PK model accounted for this effect (36). Therefore, albumin concentrations during treatment or drug-drug interactions do not explain the decrease in the intracellular-to-plasma bedaquiline and M2 ratios in HIV-positive individuals. Nevirapine has no clinically significant interaction with bedaquiline and M2 (36).

Our study has several limitations. Intracellular concentrations within PBMCs may not reflect the concentrations at the site of disease, and their rapid turnover may underestimate measure of drug accumulation in tissues. We selected PBMCs as surrogate cells because it was not feasible to perform bronchoscopies and collect alveolar macrophages. Use of PBMCs, however, provided a less invasive way to obtain the intracellular pharmacokinetic information on bedaquiline and M2 in these study participants, and this approach has been used successfully in similar studies of other drugs (37–39). Additionally, we measured only total intracellular drug concentration. For some medications, the free fraction—unbound by phospholipids—is a better measure of pharmacologically active drug, but recent evidence from *in vitro* studies suggests that bedaquiline accumulates in host cell lipid droplets and that this lipid binding may, in fact, aid in transfer of the drug to host organelles (40). This may indicate that the total drug concentration better reflects the concentration of active drug within the cell. Sampling of individuals across occasions was unbalanced, largely because participants were reluctant to return for later study visits, but this is less problematic with a modeling approach than with more traditional approaches. We obtained few samples following bedaquiline treatment completion, and this limits our precision in describing the decay of plasma/intracellular concentrations during the continuation phase of therapy. Some notable assumptions were made during the pharmacokinetic modeling. We assumed that intracellular-plasma distribution is nearly instantaneous, as estimating it was not possible. The intracellular-plasma distribution time may be longer in reality. This is expected to have small consequences for bedaquiline since total intracellular exposure is not affected and should not impact M2 at all given that the M2 pharmacokinetic profile in plasma over a dosing interval is almost flat. We also assumed that the maximum time effect on the intracellular-plasma accumulation ratio is at 2 months after treatment start, which seems reasonable for PBMCs but may differ for other cell types. Despite these limitations, this study is the first, to our knowledge, to quantify the intracellular concentrations of bedaquiline and M2 and to characterize the relationship with plasma concentrations.

In conclusion, we have shown that the concentration of bedaquiline in PBMCs is comparable to the plasma concentration during treatment and that intracellular concentrations of M2 are much higher than those of bedaquiline. Studies examining the correlation between intracellular concentrations and clinical outcomes, both efficacy and toxicity, of patients on bedaquiline will be an important next step to determine the significance of the current findings and to optimize bedaquiline dosing in this difficult-to-treat patient population.

## MATERIALS AND METHODS

### Study population.

We conducted a prospective observational study to measure the intracellular concentrations of bedaquiline in PBMCs. We enrolled a subset of participants from the PROBeX study—an observational cohort study of patients in South Africa with RR-TB on bedaquiline-containing regimens (41). Bedaquiline was dosed at 400 mg daily for the first 14 days, followed by 200 mg three times weekly for an additional 22 weeks. Consecutive substudy participants were recruited from a single center in Cape Town. Eligible participants were over the age of 18 years, had a known HIV test result, and had confirmed MDR- or XDR-TB.

### Data collection.

Consenting participants underwent intensive pharmacokinetics sampling at month 2 and sparse pharmacokinetics sampling at months 1, 2, and 6 of bedaquiline treatment. Sparse sampling was also performed 3 and 6 months after bedaquiline treatment (i.e., months 9 and 12). During the intensive pharmacokinetics sampling, blood was drawn predose and at 1, 2, 3, 4, 5, 6, and 24 h after medication was taken with food, which consisted of brown bread and peanut butter and observed bedaquiline administration. Blood draws were performed through a peripheral intravenous catheter placed for the duration of the first day of the visit. Samples were collected into 10-ml K3EDTA Vacutainer tubes (Becton, Dickinson, Franklin Lakes, NJ) and centrifuged (1,500 × *g* for 10 min) within 2 h of collection. At least 1.5 ml of plasma was pipetted into polypropylene tubes and frozen at −80°C. These procedures were done on-site. Frozen plasma was then transported to the Division of Clinical Pharmacology at the University of Cape Town for storage at −80°C until analysis.

Additional blood samples were collected in EDTA tubes at predose and 4 to 6 h and 24 h postdose for PBMC collection. Those samples were handed immediately to a courier from BioAnalytical Research Corporation (BARC SA), a commercial research laboratory that performed the PBMC isolation, washing, counting, and storage in liquid nitrogen (described below). Frozen PBMCs were delivered to the Clinical Pharmacology Laboratory at the University of Cape Town for analysis.

During the sparse pharmacokinetic sampling, single predose plasma and PBMC samples were collected at months 1, 2, and 6 of bedaquiline treatment and at 3 months and 6 months after bedaquiline treatment discontinuation (i.e., months 9 and 12). Sparse plasma and PBMC samples were processed and stored in the same manner as the intensive samples mentioned above.

Demographic and clinical data were obtained from participants at the time of the pharmacokinetic visit and at other visits as part of the parent study procedures. These data included HIV status, bedaquiline dose and duration, concomitant antituberculosis drugs and antiretrovirals, and most recent serum creatinine and albumin. The time of administration of bedaquiline and other antituberculosis drugs was also recorded.

### Ethics.

The study was approved by the Human Research Ethics Committees of the University of Cape Town (HREC reference number 286/2019), Albert Einstein College of Medicine (2014-4348), and Emory University (00081364). All participants provided written informed consent.

### Materials and chemicals.

The reference material bedaquiline and the bedaquiline-d6 internal standard were obtained from Toronto Research Chemicals (North York, Canada). The metabolite M2 and M2-d3C^13^ internal standard were donated by Janssen Pharmaceutical NV (Beerse, Belgium). Ficoll (an aqueous solution with a density of 1.077 ± 0.001 g/ml), RPMI medium, phosphate-buffered saline (PBS), trypan blue, liquid chromatography-tandem mass spectrometry (LC-MS/MS)-grade methanol (MeOH), fetal bovine serum, dimethyl sulfoxide (DMSO), and formic acid were obtained from Sigma, South Africa. LC-MS/MS-grade acetonitrile (ACN) was purchased from Honeywell, South Africa.

### Plasma assay.

The plasma assay consisted of protein precipitation extraction, followed by high-performance liquid chromatography (HPLC) with MS/MS detection. The extraction procedure was followed by LC separation using an Atlantis T3 C_18_ (3 μm, 100 mm by 2.1 mm) analytical column with a total run time of 7.5 min. An AB Sciex API 4000 mass spectrometer at unit resolution in the multiple reaction monitoring (MRM) mode was used to monitor the transition of the protonated precursor ions *m/z* 555.2, *m/z* 561.2, *m/z* 541.1, and *m/z* 545.2 to the product ions *m/z* 229.2, *m/z* 64.2, *m/z* 480.2, and *m/z* 480.2 for bedaquiline, bedaquiline-d6, M2, and M2-d3C^13^, respectively. Electrospray ionization (ESI) was used for ion production. The calibration curves fitted quadratic (weighted by 1/*x*) regressions based on peak area ratios over the ranges of 20.0 to 5,000 ng/ml for bedaquiline and 10.0 to 500 ng/ml for the metabolite M2. The combined percent accuracy and precision statistics of the low-, medium-, and high-quality control samples of bedaquiline and M2 during sample analysis were between 95.1% and 100.1%, 4.2%, and 7.7%, respectively.

### PBMC assay.

**(i) PBMC isolation from whole blood.** Whole blood was collected into blood collection tubes containing K3EDTA. The blood was centrifuged at 400 × *g* for 10 min at room temperature within 1 h of blood collection and plasma discarded. The blood was aseptically diluted with an equivalent volume of sterile PBS (1:1). Fifteen milliliters of Ficoll was transferred into 50-ml Falcon tubes. The diluted blood was then gently layered up to approximately 30 ml. The blood and the Ficoll were centrifuged at 800 × *g* for 25 min at a temperature between 18 and 22°C with brakes off. The tubes from the centrifuge were transferred to a rack, ensuring that the PBMC interface was not disturbed. The caps were gently removed and the PBMC layer was transferred into an appropriately labeled 50-ml tube and made up to 40 ml with cold PBS. The remainder of the isolation was performed on ice to prevent drug efflux. The samples were centrifuged at 300 × *g* and 4°C for 10 min. The supernatant was discarded, and the cells were resuspended by gentle mixing. Cold RPMI medium containing 2% fetal bovine serum (FBS) was added to the tubes and centrifuged at 300 × *g* for 10 min at a temperature of 4°C. The supernatant was discarded, and the cell pellet was resuspended completely in 1 ml of cold RPMI medium (containing 10% FBS) to calculate number of cells isolated per milliliter. For counting, a small volume of the resuspended cell pellet was diluted with trypan blue (1:5). An automated cell counter (508 BR04688; Bio-Rad) was used to count the cells.

### (ii) Storage of PBMCs in liquid nitrogen.

During storage of viable PBMC samples, an equal volume of freezing medium (20% DMSO in FBS) was added to the cells and gently swirled to mix. Volumes of 1 ml of the cells were transferred with freezing medium into appropriately labeled cryogenic vials which were transferred into a freezing container (Mr. Frosty) and kept at −80°C overnight. The cryogenic vials were then transferred to a liquid nitrogen container for long-term storage.

### (iii) Preparation of PBMC samples for LC-MS/MS analysis.

Viable PBMC samples were removed from the liquid nitrogen container, placed on ice, then allowed to thaw in a water bath (37°C) for approximately 3 min, and diluted with RPMI medium plus 10% FBS (PBMC suspension: RPMI medium plus 10% FBS [1:9]). The samples were then centrifuged at 400 × *g* and 4°C for 10 min. The supernatant was discarded. The cell pellets were further washed and resuspended with 1 ml of RPMI medium containing 10% FBS and gently mixed. The samples were centrifuged at 400 × *g* and 4°C for 10 min. The supernatant was discarded, and the cell pellet was lysed with 500 μl of cold lysate (MeOH-deionized water, 70:30 [vol/vol]) solution. The lysed cells were vortexed for 1 min, sonicated for 5 min, and left for 1 h at room temperature to complete the lysis process.

### (iv) Bedaquiline and M2 extraction from PBMCs and LC-MS/MS.

The PBMC assay consisted of solid-phase extraction followed by HPLC with MS/MS detection. The extraction procedure was followed by LC separation using an Agilent Poroshell 120 (2.7 μm, 4.6 mm by 50 mm) analytical column with a total run time of 6.0 min. An AB Sciex API 5500 mass spectrometer at unit resolution in the MRM mode was used to monitor the transition of the protonated precursor ions *m/z* 554.9, *m/z* 561.0, *m/z* 540.9, and *m/z* 544.9 to the product ions *m/z* 58.1, *m/z* 64.1, *m/z* 480.0, and *m/z* 480.0 for bedaquiline, bedaquiline-d6, M2, and M2-d3C^13^, respectively. Electrospray ionization was used for ion production. The calibration curves fitted quadratic (weighted by 1/*x*) regressions based on peak area ratios over the ranges of 2.5 to 200 pg/million cells for bedaquiline and 12.5 to 1,000 pg/million cells for M2. The combined accuracy and precision statistics of the low-, medium-, and high-quality controls of bedaquiline and M2 during sample analysis were between 96.3% and 99.2%, 1.0%, and 5.6%, respectively.

### Intracellular drug concentration.

To express the final intracellular concentration of a drug per cell, the following equation was adopted with a median PBMC volume of 272 fl (42):

$$\text{Intracellular concentration}\left(\text{ng/ml}\right)\;=\;\frac{\text{total amount of drug}}{\text{PBMC volume }\times \text{ total cell number/ml}}$$

### Data analysis.

The Shapiro-Wilks normality test was used to test the data for normality. Summary statistics were performed to provide a general description of the sample concentrations during and after bedaquiline treatment. The Wilcoxon matched-pairs signed-ranked test was used to test the difference in median bedaquiline and M2 concentrations in plasma and intracellular concentrations. Noncompartmental analysis was used to estimate the AUC, *C*_max_, and *C*_min_ from the intensive pharmacokinetics data at month 2. A *P* value of less than 0.05 was considered statistically significant.

For the population pharmacokinetic analysis, the plasma pharmacokinetic data were fitted without parameter reestimation using a previously established model for bedaquiline and M2 which included models describing the change in total body weight and albumin over time (18). We incorporated the known negative effect of concomitant lopinavir-ritonavir treatment on bedaquiline and M2 clearances in the plasma model by fixing the effect sizes to previously reported values (36). Individual predictive performance was assessed visually to check if the plasma concentrations were described well by the model. The individual plasma pharmacokinetic parameters were used as input to the intracellular model. Bedaquiline and M2 concentrations were converted to molar units and log transformed. The intracellular drug penetration was described using effect compartment models in which the intracellular-plasma equilibration half-life and accumulation ratio were estimated (43–45). The following equation described the intracellular penetration of bedaquiline and M2:

$$\frac{dC_{\text{intra}}}{\mathrm{dt}}=k_{\text{plasma}-\text{intra}}\times \left({\text{ACR}}_{\text{intra}}\times C_{\text{plasma}}-C_{\text{intra}}\right)$$

$$k_{\text{plasma}-\text{intra}}=\frac{\text{ln2}}{\text{HL}}$$

where *C*_intra_ is the intracellular concentration, *k*_plasma-intra_ is the time rate constant for the transfer from the plasma into the cells, ACR_intra_ is the intracellular-plasma accumulation ratio, *C*_plasma_ is the predicted concentration of bedaquiline or M2 in plasma, ln2 is the natural logarithm of 2, and HL is the equilibration half-life between the plasma and intracellular compartments. Interindividual variability in the intracellular-plasma accumulation ratio was investigated and was assumed to be log-normally distributed. Residual variability was included using a proportional model. Following the development of the structural and stochastic models, covariate effects of age, sex, race, total body weight, and HIV status on the intracellular-plasma accumulation ratio were explored. Continuous covariates were normalized to the median value in the population and tested in the model using a linear relationship. Covariate relationships were explored with the same and separate effects on bedaquiline and M2 accumulation ratios. Covariate relationships were included in a stepwise manner based on an objective function value associated with a significance level of a *P* value of <0.01 starting with the relationship with the largest drop in objective function value. Confidence intervals (CI) of the pharmacokinetic parameters were determined using the sampling importance resampling (SIR) routine (46, 47). For the SIR, the 2-fold-inflated covariance matrix from NONMEM’s covariance step was used as a starting point and the default settings were used for the number of iterations, samples, and resamples.

Stata 15 software (StataCorp, College Station, TX) was used to perform noncompartmental pharmacokinetic analysis, summarize the data, and perform statistical tests. For the population pharmacokinetic analysis, R version 3.4.3 was used for data management and plotting (48). Model development was performed using the nonlinear mixed-effects modeling program NONMEM version 7.4 with Pirana as an interface (49, 50). PsN version 4.7 was used for performing visual predictive checks and the SIR procedure (46, 47, 50). The visual predictive checks were performed using 1,000 simulations.
