# Supplementary material for: Relationship between Plasma and Intracellular Concentrations of Bedaquiline and Its M2 Metabolite in South African Patients with Rifampin-Resistant Tuberculosis
Source: Antimicrob Agents Chemother. 2021 Oct 18;65(11):e02399-20. doi: 10.1128/AAC.02399-20 (PMC8522761; doi:10.1128/AAC.02399-20)
Supplement: Supplemental file 1 — Supplemental material. Download AAC.02399-20-s0001.pdf, PDF file, 0.4 MB [file aac.02399-20-s0001.pdf]

Relationship between plasma and intracellular concentrations of bedaquiline and its M2 metabolite in South African patients with rifampin-resistant TB

Running title: Bedaquiline, M2, plasma and cells

Precious Ngwalero, BPharm, MSc[1*]; James C.M. Brust, MD[2*]; Stijn W. van Beek, MSc[3]; Sean Wasserman, MBChB[4]; Gary Maartens, MBChB, MMed[1,4]; Graeme Meintjes, MBChB, PhD[4]; Anton Joubert, BSc Hon[1]; Jennifer Norman MSc[1]; Sandra Castel PhD[1]; Neel R. Gandhi, MD[5]; Paolo Denti, PhD[1]; Helen McIlleron, MBChB, PhD[1,4]; Elin M. Svensson, PhD[3,6]; Lubbe Wiesner, PhD[1]

[1]Division of Clinical Pharmacology, Department of Medicine, University of Cape Town, Cape Town, South Africa

[2]Albert Einstein College of Medicine & Montefiore Medical Center, Bronx, NY, USA

[3]Department of Pharmacy, Radboud Institute for Health Sciences, Radboudumc, Nijmegen, The Netherlands

[4]Wellcome Centre for Infectious Diseases Research in Africa, Institute of Infectious Disease and Molecular Medicine, and Department of Medicine, University of Cape Town, Cape Town, South Africa

[5]Rollins School of Public Health and Emory School of Medicine, Emory University, Atlanta, GA, USA

[6]Department of Pharmacy, Uppsala University, Uppsala, Sweden

*Contributed equally

Correspondence: #Address correspondence to: Lubbe Wiesner, lubbe.wiesner@uct.ac.za

Figure S1. Observed versus population predicted intracellular bedaquiline and M2 concentrations

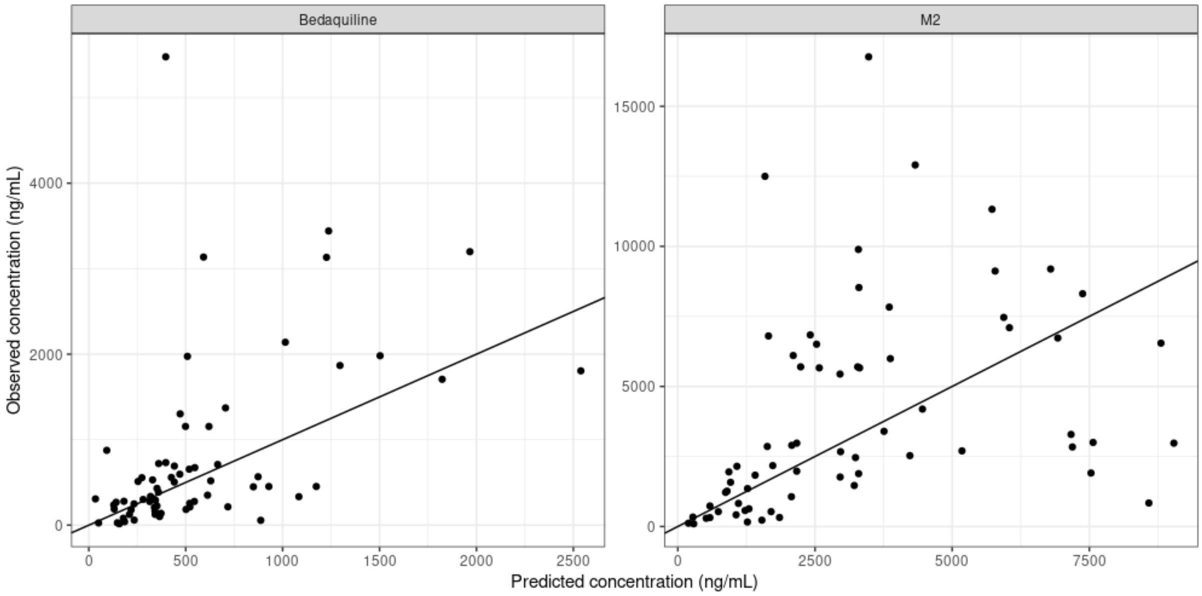

Figure S2. Observed versus individual predicted intracellular bedaquiline and M2 concentrations

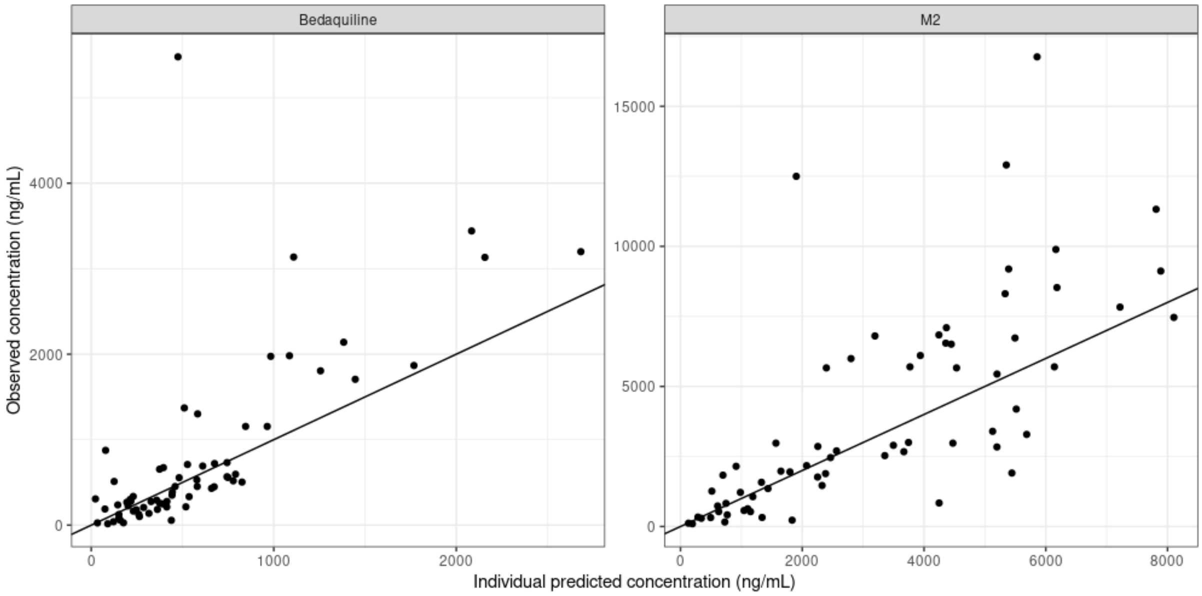

Figure S3. Conditionally weighted residuals versus time after start of bedaquiline treatment

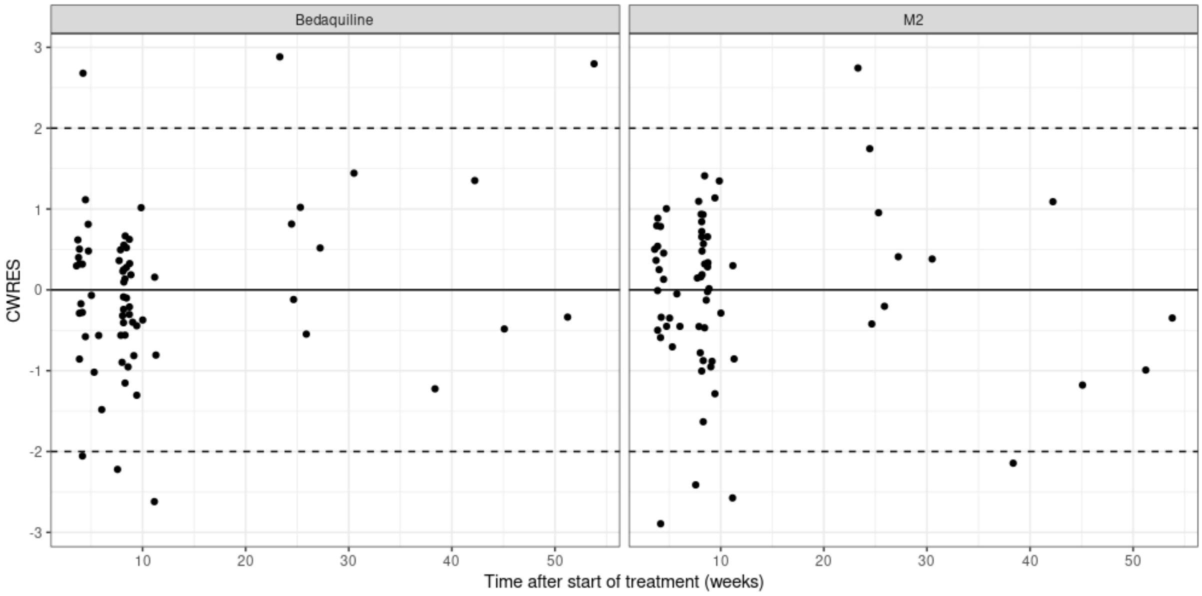

Figure S4. Conditionally weighted residuals versus time after dose

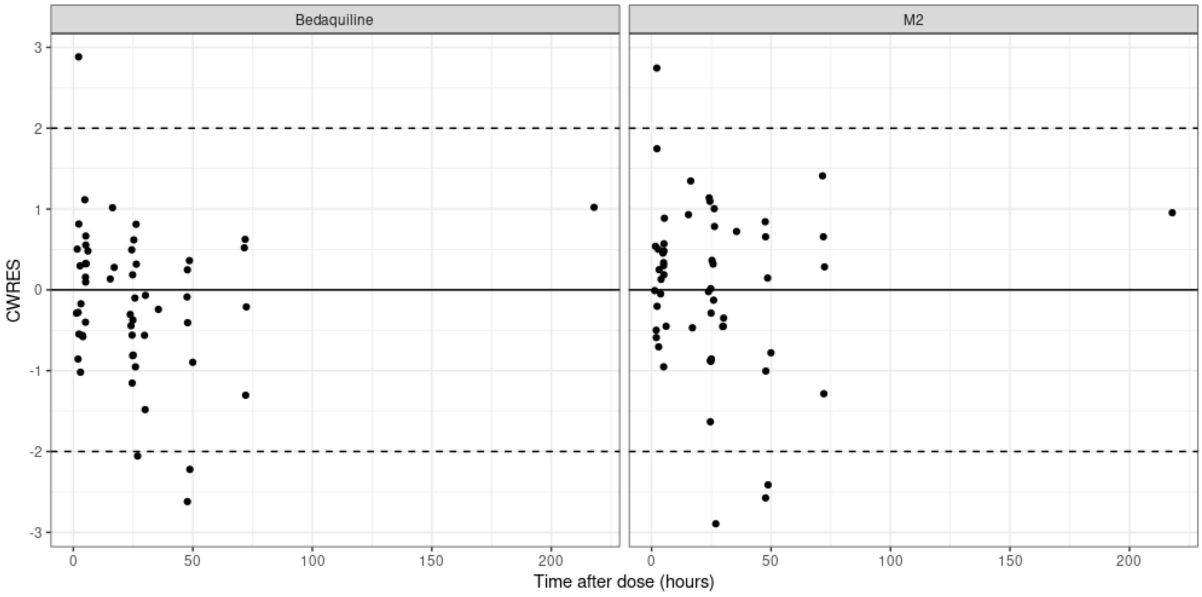

Figure S5. Conditionally weighted residuals versus population predicted bedaquiline and M2 concentrations

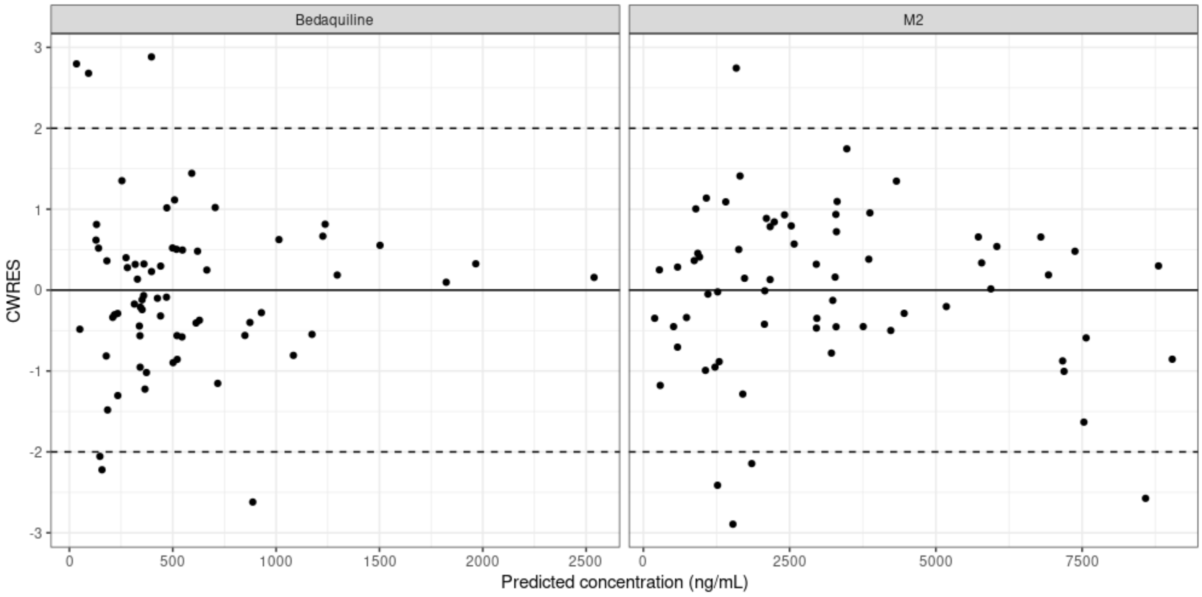

Figure S6. Visual predictive checks for intracellular bedaquiline (top) and M2 (bottom)

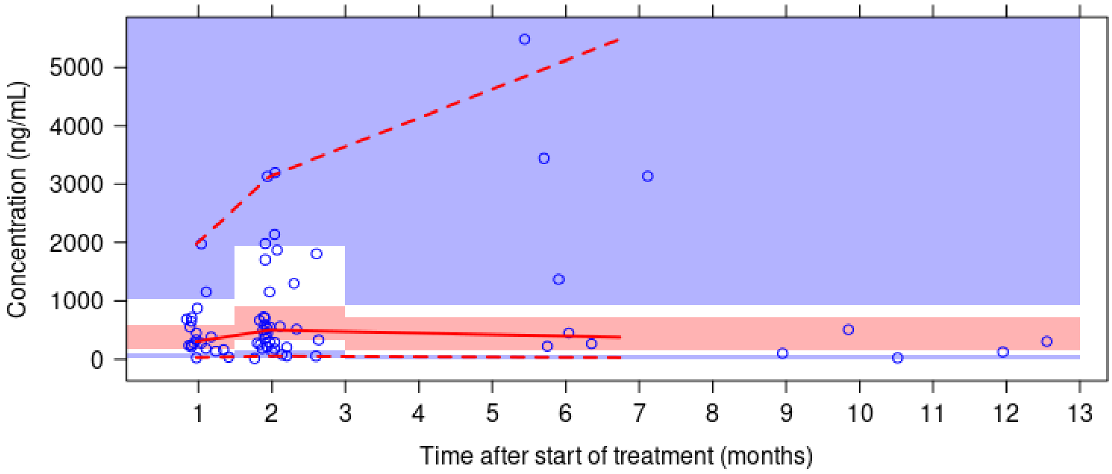

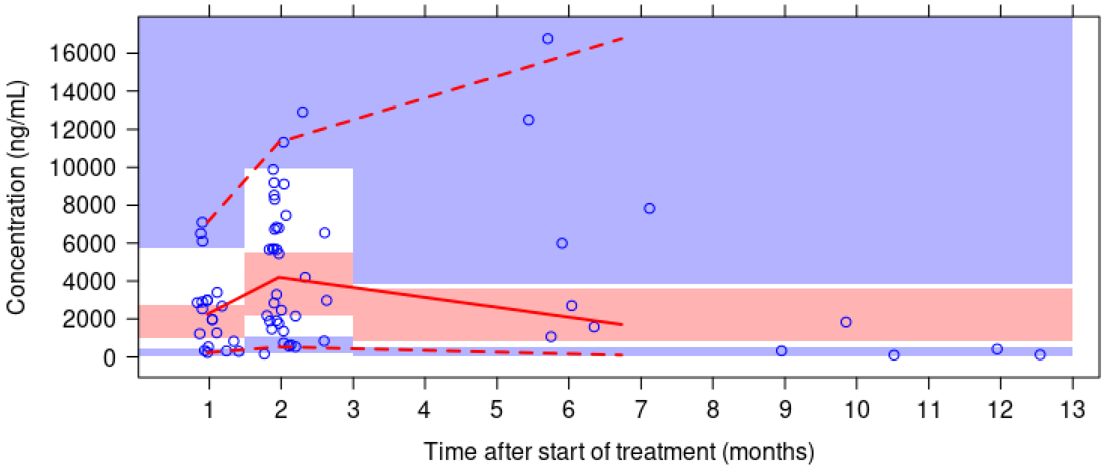

Code for the intracellular part of the pharmacokinetic model

```
$PROBLEM BDQ and M2 INTRACELLULAR PK

$INPUT ID TIME TAD DV AMT EVID II ADDL ALB SEX WT HT AGE FLAG HIV RACE L2 ET1 ET2 ET3 ET4
ET5 ET6 ET7 ET8 ET9 ET10 ET11 ET12 ET13 ET14 ET15 ET16 ET17 ET18 ET19

$DATA Dataset_including_individual_plasma_PK_parameters.csv IGNORE=@

$SUBROUTINE ADVAN13 TOL=6

$MODEL NCOMPARTMENTS=10
COMP=(DEPOT DEFDOSE)
COMP=(BDQC)
COMP=(BDQPERI1)
COMP=(BDQPERI2)
COMP=(M2)
COMP=(TRANSI1)
COMP=(TRANSI2)
COMP=(ALBUMIN)
COMP=(BDQINTRA)
COMP=(M2INTRA)

$PK

; HIV covariate effect on the intracellular-plasma accumulation ratio
HIVACR = 1 + HIV*THETA(29)

; Intracellular-plasma equilibration half-life
HLBDQ  = THETA(27)
HLM2   = THETA(27)

; Intracellular-plasma accumulation ratios at month 1500h (~2 months)
ACRBDQ = THETA(25) * HIVACR * EXP(ETA(1))
ACRM2  = THETA(26) * HIVACR * EXP(ETA(1))

; Intracellular-plasma accumulation ratios at the start of BDQ treatment
MINACRBDQ      = ACRBDQ*THETA(28)
MINACRM2   = ACRM2*THETA(28)

$DES

; Time effect on the intracellular-plasma accumulation ratios
T2 = T
IF(T2.GT.1500) T2 = 1500
ACRM2T = (ACRM2-MINACRM2)*(T2/1500) + MINACRM2
ACRBDQT = (ACRBDQ-MINACRBDQ) *(T2/1500) + MINACRBDQ

; Differential equations for the intracellular compartments
DADT(9) = LOG(2)/HLBDQ*( (ACRBDQT)*A(2)/V - A(9) )
DADT(10)= LOG(2)/HLM2*( (ACRM2T)*A(5)/VM2 - A(10) )
```

```
$ERROR

; FLAG 1 = BDQ PK, 2 = M2 PK, 3 = Albumin, 4 = Body weight, 5 = intra BDQ PK, 6 = intra M2 PK
IF(FLAG.EQ.5) IPRED = LOG(A(9))
IF(FLAG.EQ.6) IPRED = LOG(A(10))

; Error additive on log scale for PK
IF(FLAG.EQ.5) Y = IPRED + EPS(5)
IF(FLAG.EQ.6) Y = IPRED + EPS(6)

$THETA
(0, 0.8)  ; 25 MAX ACRBDQ
(0, 16)  ; 26 MAX ACRM2
(0,0.017) FIX   ; 27 HLBDQ
(0,0.3)  ; 28 FACTOR MIN ACR
(-0.5)   ; 29 HIV-ACR

$OMEGA
0.01  ; 1 BSVACR

$SIGMA
1 ; 5 Prop error BDQ intra
0.8 ; 6 Prop error M2 intra

$ESTIMATION METHOD=1 MAXEVAL=9999 PRINT=1 SIGL=9 NSIG=3 NOABORT INTERACTION
```
